# Robust Vehicle Detection under Various Environmental Conditions Using an Infrared Thermal Camera and Its Application to Road Traffic Flow Monitoring

**DOI:** 10.3390/s130607756

**Published:** 2013-06-17

**Authors:** Yoichiro Iwasaki, Masato Misumi, Toshiyuki Nakamiya

**Affiliations:** Department of Information Engineering, Graduate School of Industrial Engineering, Tokai University, 9-1-1 Toroku, Higashi-ku, Kumamoto 862-8652, Japan; E-Mails: 2bijm001@mail.tokai-u.jp (M.M.); nakamiya@tokai.ac.jp (T.N.)

**Keywords:** vehicle detection, infrared thermal images, various environmental conditions, traffic monitoring, thermal energy reflection, Intelligent Transportation Systems (ITS)

## Abstract

We have already proposed a method for detecting vehicle positions and their movements (henceforth referred to as “our previous method”) using thermal images taken with an infrared thermal camera. Our experiments have shown that our previous method detects vehicles robustly under four different environmental conditions which involve poor visibility conditions in snow and thick fog. Our previous method uses the windshield and its surroundings as the target of the Viola-Jones detector. Some experiments in winter show that the vehicle detection accuracy decreases because the temperatures of many windshields approximate those of the exterior of the windshields. In this paper, we propose a new vehicle detection method (henceforth referred to as “our new method”). Our new method detects vehicles based on tires' thermal energy reflection. We have done experiments using three series of thermal images for which the vehicle detection accuracies of our previous method are low. Our new method detects 1,417 vehicles (92.8%) out of 1,527 vehicles, and the number of false detection is 52 in total. Therefore, by combining our two methods, high vehicle detection accuracies are maintained under various environmental conditions. Finally, we apply the traffic information obtained by our two methods to traffic flow automatic monitoring, and show the effectiveness of our proposal.

## 1. Introduction

Vision-based traffic measurement systems have the advantage of measuring those domains which cannot be measured by conventional vehicle detectors: vehicle positions and vehicle movements in multilane traffic. Therefore, by using vision-based traffic measurement systems, we can perform both an automatic traffic monitoring system designed to find traffic incidents without time delay and an optimal traffic signal control method designed to reduce traffic jams.

The present level of vision-based traffic measurement systems research is enough to detect vehicles around the clock [1–3]. However, there are some defects which are described below. Many daytime vehicle detection methods have been developed, in which a method to separate occluded vehicles has been proposed in order to increase detection accuracy [4]. However, it is difficult to apply these methods to nighttime detection. Yoneyama *et al.* [2] have pointed out that most of the daytime detection methods lose their accuracy when they are directly applied to nighttime detection. Therefore, generally adopted methods detect headlights or taillights of vehicles at nighttime, and use two algorithms separately for daytime and nighttime detection [1–3]. These nighttime detection algorithms cannot measure each vehicle position in queues under heavy traffic because the vehicular gaps must be long to reveal fully the pairs of headlights or taillights. Shadows cast by vehicles in daytime impair vehicle detection. Therefore, complicated vehicle-cast shadow elimination algorithms have also been proposed to remedy this situation [5,6].

In conventional methods of vehicle detection with visible light cameras, it is difficult to detect vehicles with high accuracy in poor visibility conditions such as fog, snow, heavy rain, and darkness. However, traffic accidents and traffic jams commonly occur under such circumstances. Therefore, it is a pressing matter for us to develop a method for detecting vehicles with high accuracy under all circumstances.

In our previous study, we proposed a method for detecting vehicle positions and their movements (henceforth referred to as “our previous method”) by using thermal images obtained with an infrared thermal camera instead of a visible light camera [7]. The thermal images are expected to detect vehicles robustly around the clock regardless of changing environments.

An infrared thermal camera is usually referred to as a passive sensor. On the other hand, active sensors are also used vehicle detection. In recent years millimeter wave radars have commonly been used as active sensors. Millimeter wave radars are not affected by poor visibility conditions, and can detect vehicles in multilane traffic with sufficient accuracy. However, radar sensors have some weaknesses. Interference among vehicle sensors and sensors on road infrastructure poses a big problem [8–10]. Interference to radio astronomy is a more sensitive issue [10]. Moreover, since radar gives limited information regarding the shape of the detected object, it is not reliable to distinguish a vehicle from other objects and obstacles [11,12]. Therefore, we use an infrared thermal camera as a passive vision sensor.

Recently, a few vehicle detection algorithms using infrared images have been proposed [13,14]. However, these algorithms cannot detect the positions of many vehicles in queues under heavy traffic, therefore, these algorithms are not useful for automatic traffic monitoring and optimal traffic signal control systems. Our experiments [7] have proved that our previous method can detect vehicles robustly in queues under heavy traffic. Our previous method uses the windshield and its surroundings as the target of pattern recognition using the Viola-Jones detector [15,16]. Some experiments in winter showed that the vehicle detection accuracy decreases because the temperatures of many windshields approximate those of the exterior, therefore, it is necessary to realize a new method without relying on this pattern recognition.

In this paper, we propose a new vehicle detection method (henceforth referred to as “our new method”). Our new method detects vehicles based on distinguishing the tires' thermal energy reflection area on the road surface from the other areas.

First, in Section 2 we will explain advantages of thermal images in vehicle detection under poor visibility conditions. Second, we will briefly explain our previous method, the experiments, and their results in Section 3. Third, we will explain our new method, the experiments, and their results in Section 4. Fourth, we will show an application of our previous method and our new method (henceforth referred to as “our two methods”) to road traffic monitoring in Section 5. Finally, we will present our conclusions in Section 6.

## 2. Advantages of Thermal Images in Vehicle Detection under Poor Visibility Conditions

The infrared thermal camera used in our detection is a TVS-500EX [17]. The frames of the infrared thermal images are transmitted to a personal computer with a 1/60 s interval through the USB 2.0 interface. The frame size of the images is 320 × 240 pixels, and each pixel has 256 gray levels. We have also used another type of infrared thermal camera, a TVS-200 [18]. Its frame rate, frame size, and number of gray levels are same as those of the TVS-500EX. The interface between the TVS-200 and a computer is IEEE1394. Therefore, our two methods detect vehicles in thermal images taken with one of the two infrared thermal cameras, the TVS-500EX and the TVS-200.

**Figure 1.**
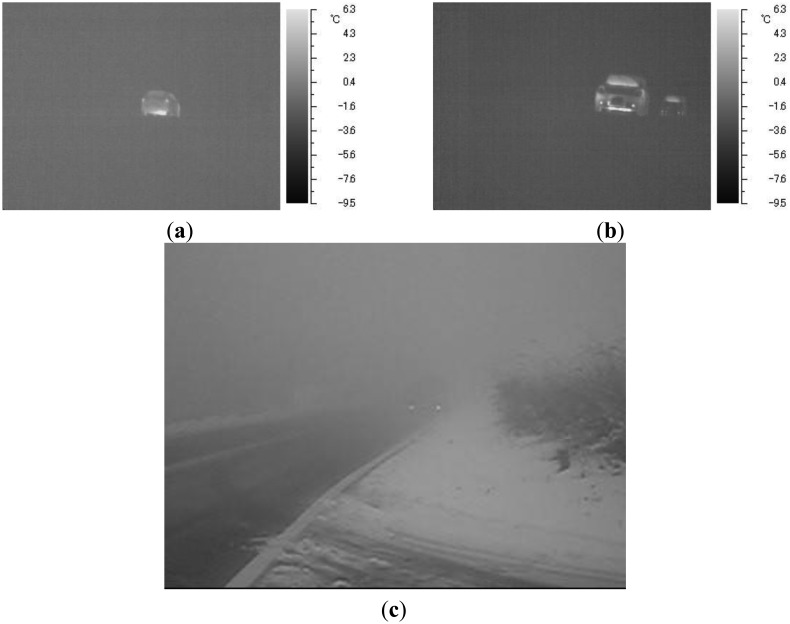
Thermal images and a visible light image in snow and thick fog: (**a**) thermal image (back side view), (**b**) thermal image (front side view), and (**c**) visible light image (front side view).

We have confirmed through our observations that the obtained thermal images are highly contrasted enough to detect vehicles, even in poor visibility conditions. We have obtained both thermal images and visible light images in snow and thick fog on a mountain road at Aso in Kumamoto, Japan. Images of moving vehicles taken with the TVS-200 infrared thermal camera are shown in Figure 1(a,b), and an image taken with a visible light camera is shown in Figure 1(c). The vehicles are clearly seen in Figure 1(a,b), while only the position of the fog lamps can be seen in Figure 1(c). The bars on the right sides in Figure 1(a,b) show the temperature scale. It was snowing at the time, and the drivers were using the windshield wipers. We investigated the temperatures of 50 points on the windshields and 50 points of the exterior of the windshields. As a result, it was determined that the temperature on the windshields is on average 3.64 °C higher than that of the exterior of the windshields. Even with such a small difference in temperature, our previous method detects two vehicles in Figure 1(b) as shown in Section 3.5. Although we did not measure the distances between the cameras and the vehicles, we already confirmed that the vehicle behind in Figure 1(b) is obviously further from the camera compared to that in Figure 1(c) when taking the images. The angle of view of the infrared thermal camera is different from that of the visible light camera. Our two methods aim for application to both an automatic traffic monitoring system and an optimal traffic signal control method. The information of upstream traffic flows at a long distance is effective for these applications. Therefore, it is important to detect vehicles further from the camera.

We have also proposed a moving vehicle detection method based on an optical flow algorithm using the TVS-200 infrared thermal camera, and detected moving vehicles in total darkness without street lights [19]. In reference [20], we can clearly see objects in an infrared thermal image taken in heavy rain. Reference [21] describes how weather conditions affect the attenuations of infrared thermal radiation from the objects. Reference [21] states as follows: even though rain drops are larger than fog droplets, their concentration is lower, and this means that rain does not scatter thermal radiation as much as fog does. Reference [21] shows that the highest attenuation occurs in thick fog as compared with four other weather conditions: urban pollution, light rain, fog, and heavy rain. The spectral range of the infrared thermal camera used in reference [21] is as same as that of the two infrared thermal cameras we used. We can say based on the physical characteristics of infrared thermal radiation that the infrared thermal cameras can be effectively used for vehicle detection under poor visibility conditions because we have confirmed that the infrared thermal camera offers images with sufficient contrast in thick fog conditions.

## 3. Our Previous Method, the Experiments, and Their Results

**Figure 2.**
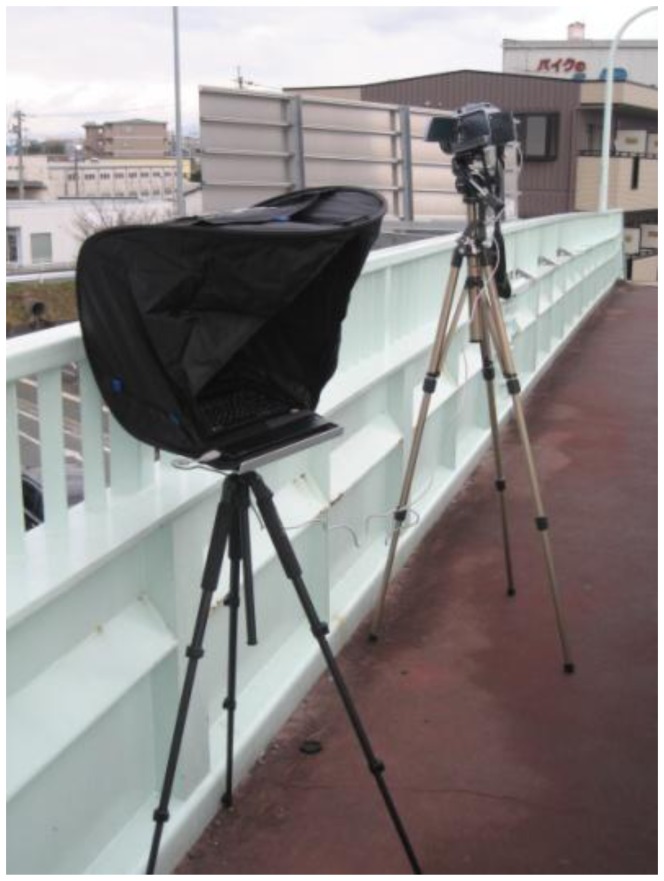
The infrared thermal camera and the notebook personal computer on a pedestrian bridge.

**Figure 3.**
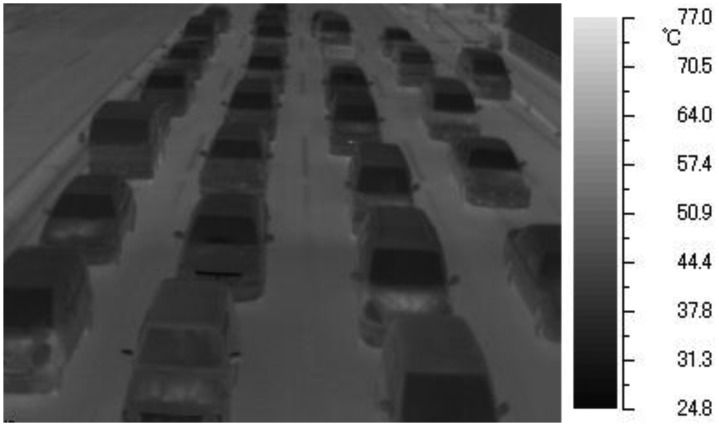
A frame of thermal images.

Figure 2 shows the TVS-500EX infrared thermal camera and the notebook personal computer to capture thermal images on a pedestrian bridge. To shield the display of the notebook personal computer against the sun, the notebook personal computer is covered with a hood. Figure 3 shows one frame of thermal images as detection target. We will explain the algorithms of our previous method in Sections 3.1–3.4, and its experiments and results in Section 3.5.

### 3.1. Spatio-Temporal Image Processing

**Figure 4.**
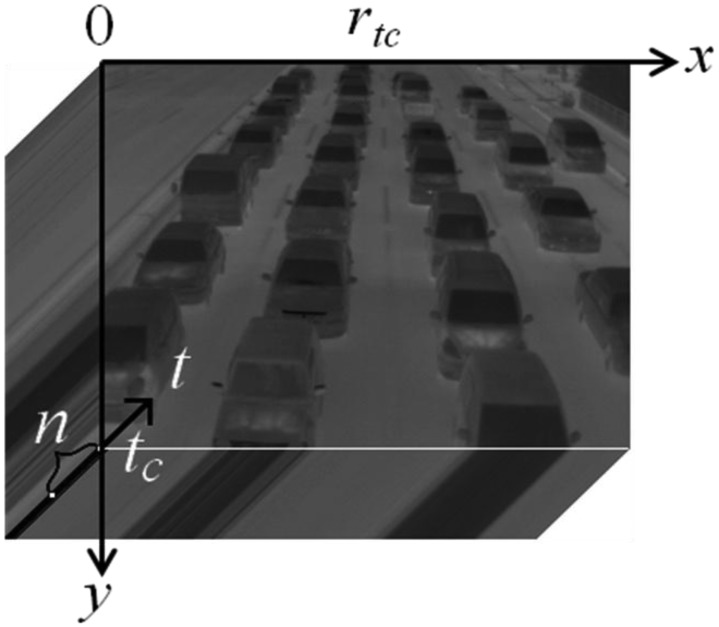
An example of spatio-temporal images.

Figure 4 shows an example of spatio-temporal images with the inscription of space-axes *xy* and time-axis *t*. The standard deviation of each pixel values for *n* frames in the past are calculated from the thermal images as follows:

(1) $$\sigma (x,y,r_{tc})=\sqrt{\frac{\sum_{k=0}^{n-1}{\left(f(x,y,r_{tc}-k)-\mu (x,y,r_{tc})\right)}^{2}}{n}}$$

where *σ* (*x*, *y*, *r_tc_*) is the standard deviation at coordinates (*x*, *y*) for *n* frames in the past from the frame *r_tc_* at the current time, *f*(*x*, *y*, *r_tc_*-*k*) is the pixel value at coordinates (*x*, *y*) in the frame *r_tc_*-*k*, and *μ* (*x*, *y*, *r_tc_*) is the mean value at coordinates (*x*, *y*) for *n* frames in the past from the frame *r_tc_*.

In this paper, the origin of the image coordinate system is located at the upper left-hand corner of the image. The standard deviations indicate the variations of pixel values along the time direction. By computing the standard deviations of all pixels in the frame, we can distinguish between the area of moving vehicles and that of the background or stopped vehicles based on *n* frames in the past. When the standard deviation is more than or equal to *sd_t_*, the pixel is actually assumed to be in the area of moving vehicles because each pixel value includes noise.

### 3.2. Vehicle Pattern Recognition Using the Viola-Jones Detector

**Figure 5.**
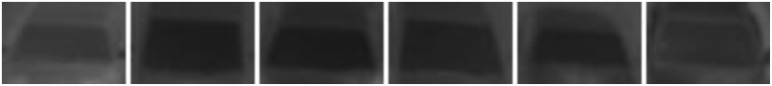
Some examples of the positive sample images.

To detect vehicles, we use the Viola-Jones detector [15]. In our vehicle pattern recognition, we use an extended algorithm proposed by Lienhart *et al.* [16] for the work presented in [15]. Each image we have collected contains the windshield and its surrounding which clearly show the front view of a vehicle as positive samples while images contain no vehicle as negative samples. Figure 5 shows some examples of the positive sample images. By using the upper part of the vehicle such as the windshield and its surroundings as the target of pattern recognition, we can make a robust detection of vehicles even when they are stopped one after another with a short distance. We have conducted training with these sample images to obtain a multistage cascade of classifiers, and finally managed pattern recognition of vehicles with the obtained multistage cascade of classifiers.

Some vehicle detection methods using the Viola-Jones detector have been proposed [22–24]. They use visible light images, and need a wider target area than used in our previous method, which means they cannot be applied to detection of stopped vehicles with short distances.

### 3.3. Correction Procedures for Misrecognition of Vehicles

In order to increase vehicle detection accuracy, the following correction procedures are applied to vehicle pattern recognition results.

When the omission of vehicle detection occurs in the vehicle pattern recognition, the omitted vehicle position is searched from the vehicle position in the previous frame by using pattern matching. In order to match the template against the traffic image with high accuracy, we have examined several matching methods. As a result, we have selected the normalized correlation coefficient matching method [25].

The size of windshield is bigger as the vehicle comes nearer to an infrared thermal camera. So, we have done the regression analysis on the relationship between *y* positions (independent variable) and the sizes of the recognition-target areas (dependent variable). The size of the recognition-target area is calculated by the obtained regression equation after substitution of *y* position. If the size of detected object is less than *S_t_*% of the calculated size, the detected object is treated as a non-recognition target.

### 3.4. Combination of Two Kinds of Information: Vehicle Positions and Their Movements

By combining the two kinds of processing (spatio-temporal image processing described in Section 3.1, and vehicle pattern recognition with the correction procedures described in Sections 3.2 and 3.3) in the same frame of images, the position of each vehicle can be specified, and its movement can be classified, too. The speed of each vehicle can be classified based on the ratio of the area of the moving vehicle in the rectangle which shows the windshield and its surroundings of vehicle. In our previous method, we have classified three categories. If the ratio is less than *a*_1_%, the vehicle is assumed to be stopped. If the ratio is more than or equal to *a*_1_% and less than *a*_2_%, the vehicle is assumed to be running at low speed. If the ratio is more than or equal to *a*_2_%, the vehicle is assumed to be running at high speed.

### 3.5. Vehicle Detection Experiments

**Figure 6.**
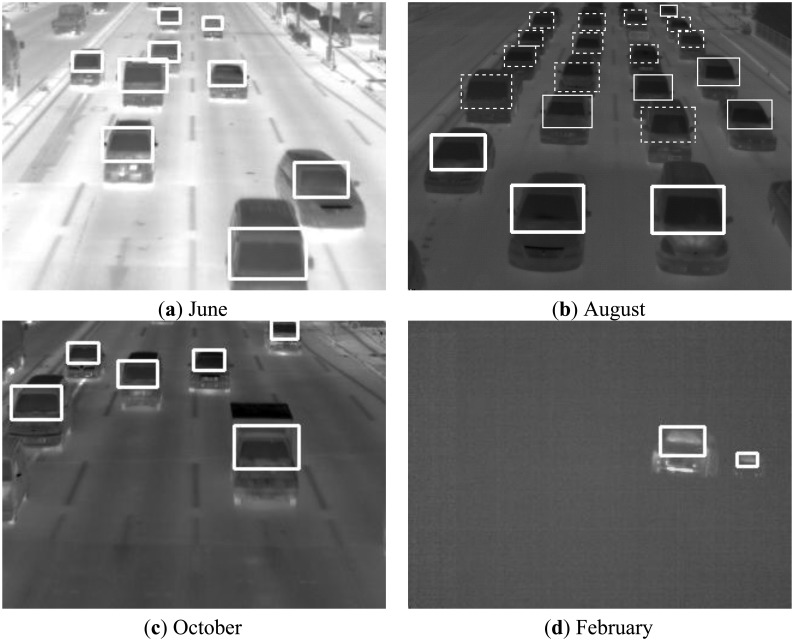
Some vehicle detection results in thermal images.

**Figure 7.**
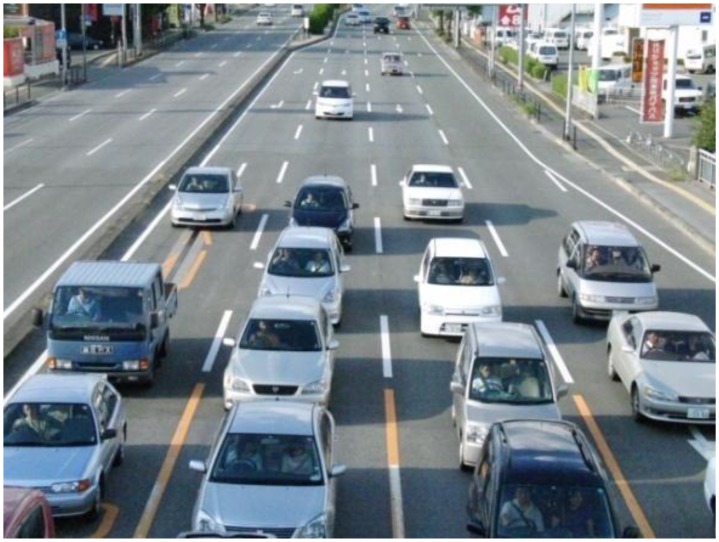
One frame of the visible light images taken in August.

The number of positive sample images and negative ones used in our experiments are 20,984 and 9,500, respectively. We have assumed in the experiments that *n*, *sd_t_*, *S_t_*, *a*_1_, and *a*_2_ are 30, 3.0, 40.0%, 10.0%, and 40.0%, respectively. Figure 6 shows some vehicle detection results in thermal images which were taken in June, August, October, and February. The dotted lines, the thin lines, and the bold lines in Figure 6 show three categories of vehicles: stopped vehicles, slow moving vehicles, and fast moving vehicles, respectively. When the thermal images were taken in August, we also took visible light images with a digital camera. Figure 7 shows one of the visible light images in which the vehicle cast shadows extend to the adjoining lanes. There is no influence of vehicle cast shadows on the vehicle detection using the thermal images. In the experiments in June, August, and October, our previous method detects 1,404 vehicles (96.2%) out of 1,460 vehicles, and the number of false detection is 35 in total. Moreover, we have used our previous method to detect a series of images taken with an infrared thermal camera under snow and thick fog in February. Two frames of the images are shown in Section 2. Our previous method detects the vehicles robustly in the 222 continuous frames. The temperature of the windshield is usually higher than that of the exterior of the windshield in winter cold days. Thus, the relationship between the two temperatures is reversed in such an environment. Our previous method can detect vehicles on cold days by simply employing the negative transformation for the original images as a preprocessing step.

## 4. Our New Method, the Experiments, and Their Results

### 4.1. The Necessity of Our New Method

We used the image data taken in August as positive and negative samples for the machine learning to develop our previous method. In summer, the temperature of the windshield is lower than that of the exterior of the windshield. This relationship continues from spring to autumn as can be seen from the images of Figure 6(a–c) in Western Japan. On the other hand, the temperature of the windshield is higher than that of the exterior in the image of Figure 6(d), which was taken in February below freezing point. For this relationship, our previous method works with high accuracy by simply employing the negative transformation for the original images as described in Section 3.5.

**Figure 8.**
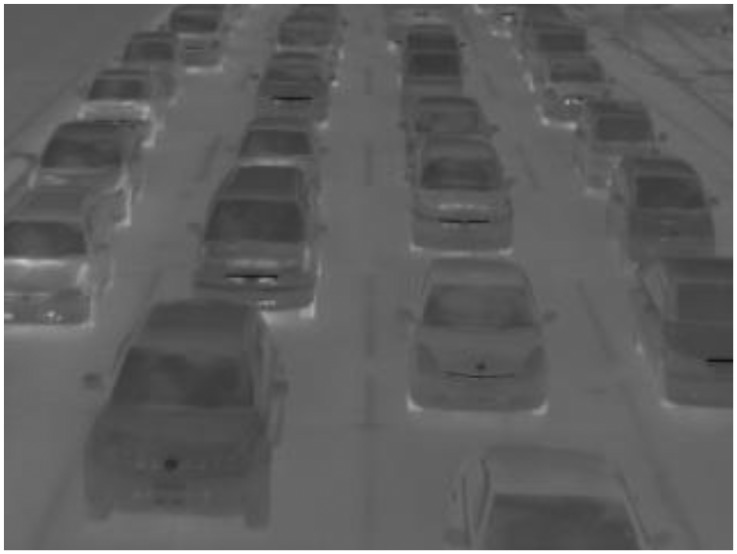
A thermal image in which the temperatures of the windshields similar to those of the exterior of the windshields.

However, in winter above freezing point, the vehicle detection accuracy of pattern recognition by the Viola-Jones detector decreases because the differences between two temperatures are reduced as shown in Figure 8. Therefore, it is necessary to realize our new method without relying on this pattern recognition. By combining our two methods, high vehicle detection accuracies will maintain under various environmental conditions.

### 4.2. A Proposed Algorithm Utilized Tires' Thermal Energy Reflection

An infrared thermal camera receives mainly two types of vehicle thermal energy: thermal emission energy obtained directly from vehicles, and vehicles' thermal energy reflected by the road surface. We have confirmed that the thermal images taken with the infrared thermal cameras offer high pixel values in the tires' thermal energy reflection area on the road surface. When the open air temperature is low like in winter, the temperature difference between the tires and the road surface is high [26]. Therefore, if the pixel values located both side of vehicles are automatically measured, and tires' thermal energy reflection area is discriminated, the vehicles can be detected.

**Figure 9.**
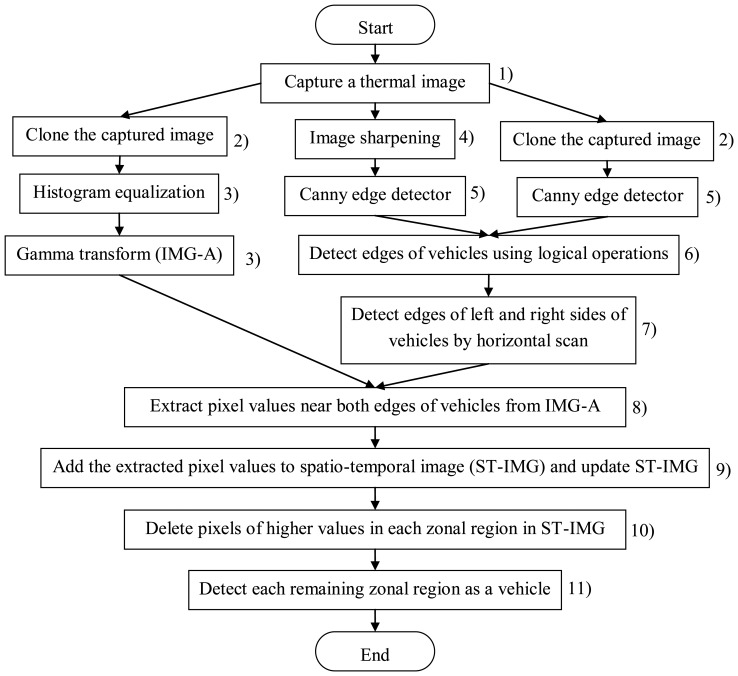
The flowchart of our new method.

We describe the algorithm of our new method using Figure 9. Each number below corresponds to that in Figure 9.

**Figure 10.**
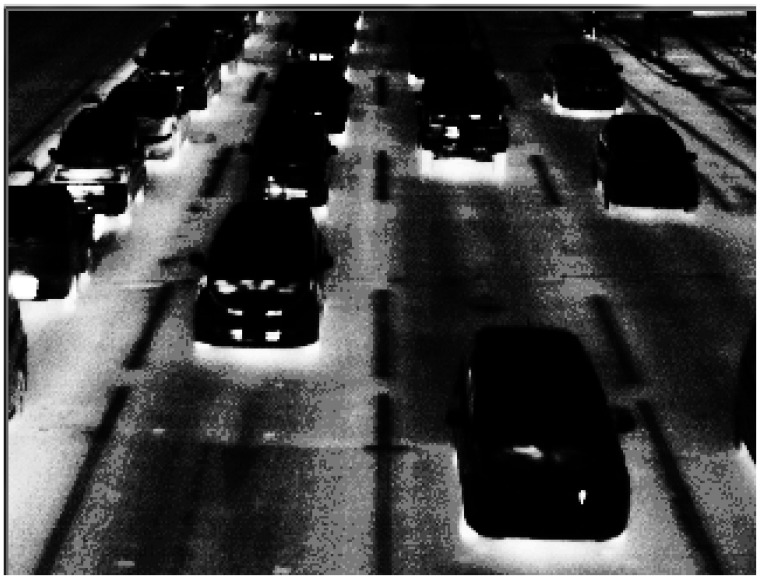
A transformed image “IMG-A”.

**Figure 11.**
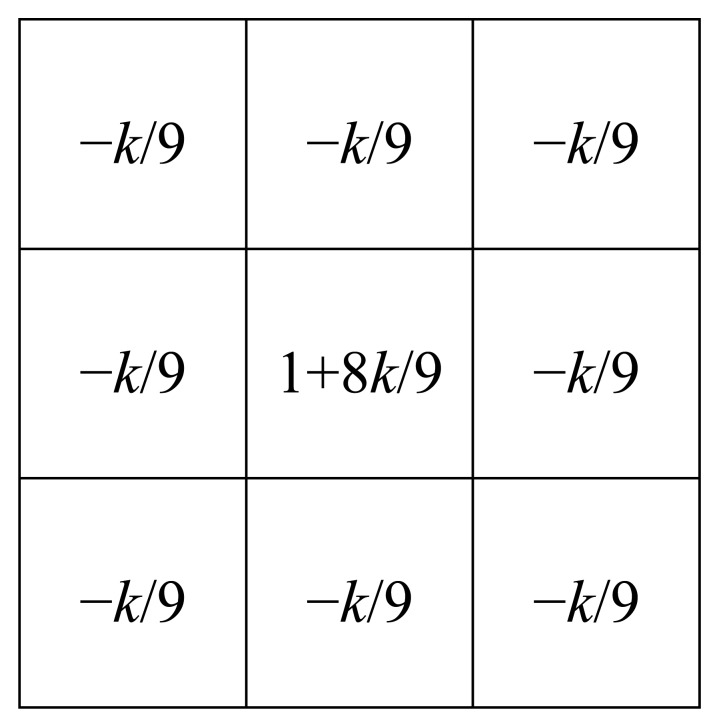
The kernel for unsharp masking.

**Figure 12.**
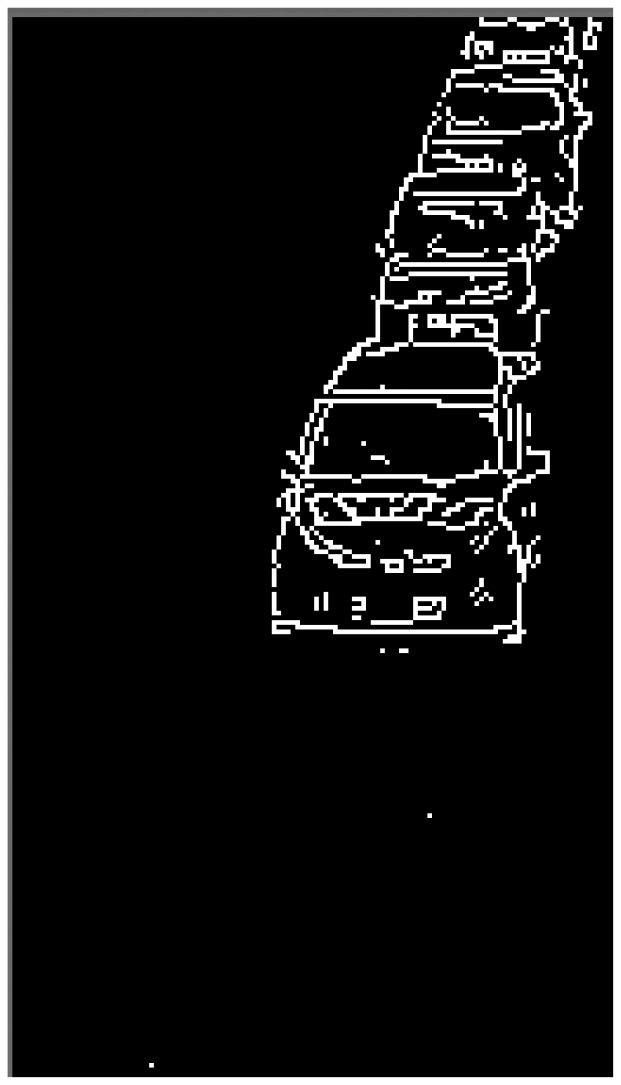
An edge image.

**Figure 13.**
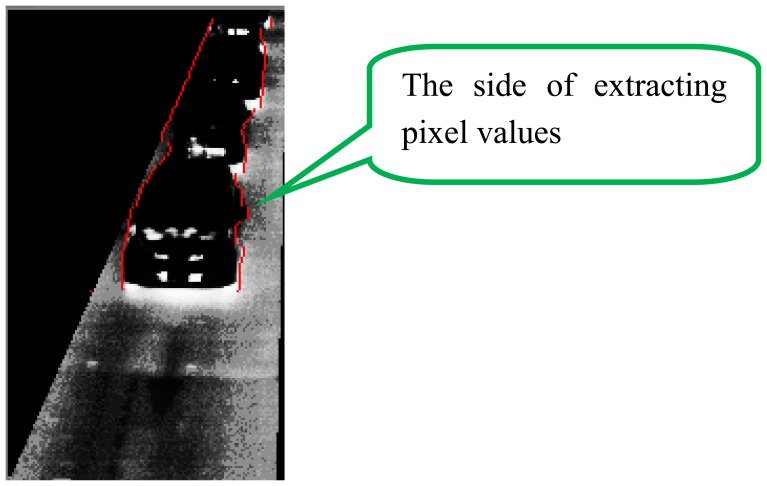
The positions shown in red lines to extract pixel values from an IMG-A.

**Figure 14.**
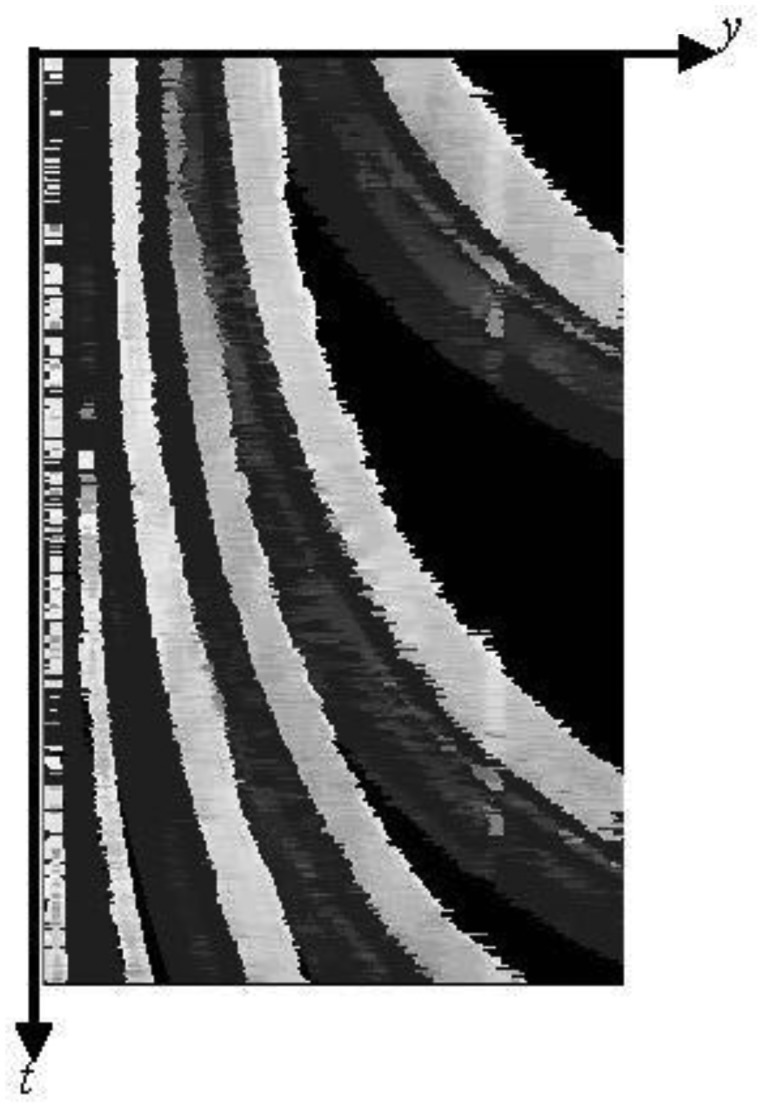
A spatio-temporal image “ST-IMG”.

**Figure 15.**
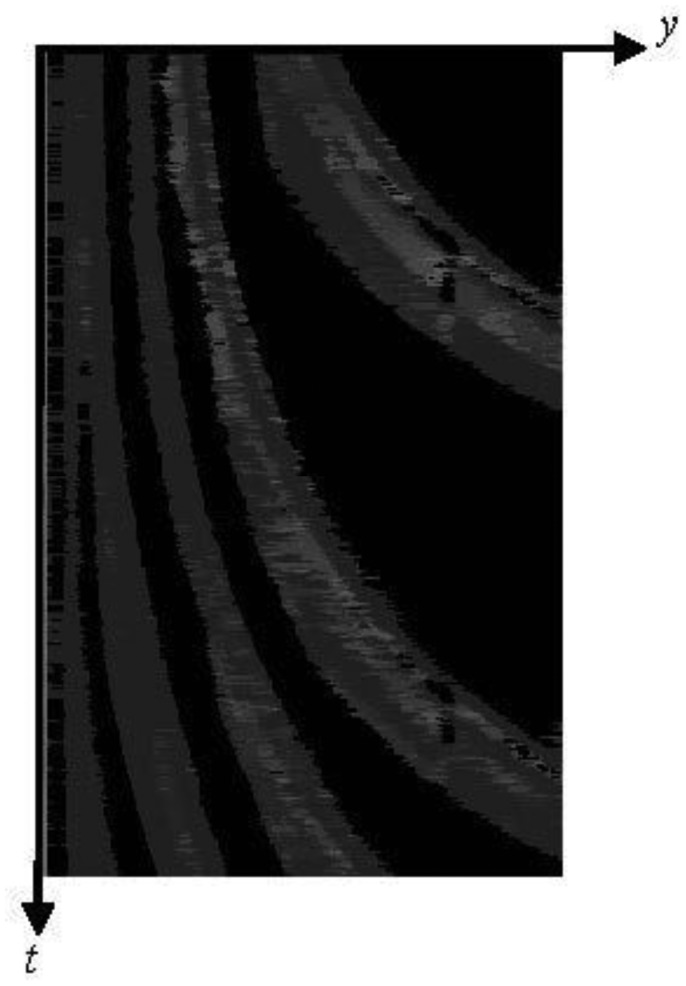
A spatio-temporal image “ST-IMG” with low pixel values only.

(1) A thermal image is captured.
(2) To do three types of image processing for the original image, the image is cloned. Then, the three same images are obtained.
(3) Histogram equalization is employed for the original image. Then, gamma transformation with *γ* is employed. By these two processes, we can obtain the image which emphasizes the tires' thermal energy reflection area. We call the transformed image as “IMG-A” in this section. An IMG-A is shown in Figure 10.
(4) Image sharpening using unsharp masking is employed for the original image. Figure 11 shows the kernel with *k* for unsharp masking.
(5) Canny edge detector with low and high thresholds, *T_L_* and *T_H_*, is employed for two types of images: sharpened image and original image.
(6) Two types of edge images are obtained. The edges of vehicles in edge original image are not completely appeared. On the other hand, the edges of vehicles in edge sharpened image are clearly appeared, but edge sharpened image contains random noise. To reduce the random noise, logical operation AND is done between two continuous edge sharpened images. After the logical operation, connected components are searched based on the remained edge pixels. Logical operation OR is done between the obtained image which contains connected components and the edge original image. Figure 12 shows an obtained edge image.
(7) The edges of left and right sides of vehicles are detected by horizontal scan.
(8) Pixel values on the outer sides of the left and right edges are extracted from IMG-A. The distance *w* between the *x*-coordinate of extracted pixel and that of edge is decided by:
(2) w=ay+1
  where *a* is the coefficient of coordinate *y*.
  Figure 13 shows the red lines which indicate the positions to extract pixel values from an IMG-A. To apply our new method to every lane, both edges for a lane can be detected. In fact, the pixel values at the side closer to the center of the road are used to detect vehicles as shown in Figure 13.
(9) The extracted pixel values are smoothed by the one-dimensional median filter of length 9, and added to the spatio-temporal image. We call the spatio-temporal image as “ST-IMG” in this section. Thus, ST-IMG is updated. Figure 14 shows a ST-IMG.
(10) The tires' thermal energy reflection exists in two areas of front and rear tires for each vehicle. Therefore, we detect the area of pixels with low values using the process described below to extract one area from each vehicle.
  The mean pixel value *μ_z_* of each zonal region in ST-IMG is computed as:
(3) $$\mu_{z}=\frac{\sum_{y\in Z}f(y,t)}{n_{Z}}$$
  where *Z* is the *y*-range of each zonal region, *f*(*y*, *t*) is the pixel value at coordinate *y* and time *t*, and *n_Z_* is the number of pixels in each zonal region.
  Then, the pixels with values of not less than *μ_Z_* are extracted, and the mean pixel value *μ_Z__*_bright_ is computed as:
(4) $$\mu_{Z\_\text{bright}}=\frac{\sum_{y\in Z\_\text{bright}}f(y,t)}{n_{Z\_\text{bright}}}$$
  where *Z*_ bright contains *y*-coordinates of pixels with values of not less than *μ_Z_* in each zonal region, and *n_Z_*__bright_ is the number of pixels with values of not less than *μ_Z_* in each zonal region.
  The pixels *g*(*y*, *t*) with low values are extracted as:
(5) $$g(y,t)=\{\begin{array}{c} \begin{array}{c} \begin{array}{cc} 0 & f(y,t)\geq \mu_{Z\_\text{bright}} \end{array}\\ \begin{array}{cc} f(y,t) & \;\text{otherwice} \end{array} \end{array} \end{array}$$
  Figure 15 shows a ST-IMG with low pixel values only.
(11) Each remaining zonal region is detected as a vehicle. Our new method repeats the process from (1) to (11).

### 4.3. Vehicle Detection Experiments

We have done the experiments using three series of thermal images taken in February for which the vehicle detection accuracies of our previous method are low. We have assumed in the experiments that *γ*, *k*, *T_L_*, *T_H_*, and *a* are 0.3, 3.0, 40, 50 and 0.01, respectively.

**Figure 16.**
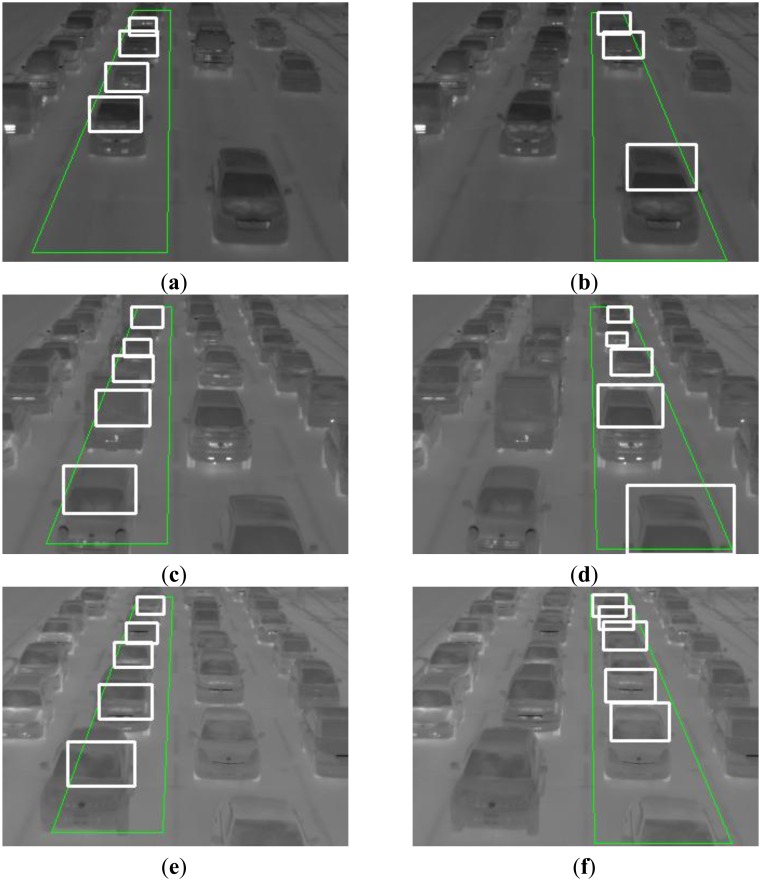
Some vehicle detection results.

Figure 16 shows six examples in detection results for the three series of thermal images. In the experiments, we used two center lanes which have high traffic volumes, and detected vehicles in each lane. The green rectangles in Figure 16 show the vehicle detection areas.

Our new method detects 1,417 vehicles (92.8%) out of 1,527 vehicles, and the number of false detection is 52 in total. Occlusion robust vehicle detection is performed because our new method uses the thermal information of outside of vehicles.

## 5. Automatic Monitoring of Traffic Flow Conditions

We have applied the traffic information obtained by our two methods to automatic traffic flow monitoring. An example of automatic traffic flow monitoring is described below.

**Figure 17.**
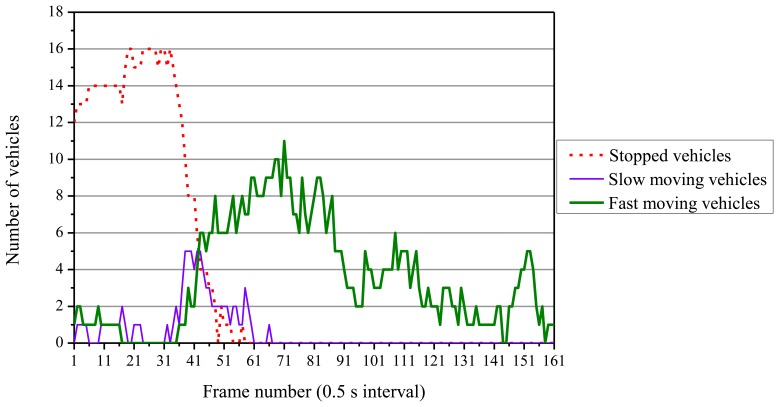
Fluctuations of the numbers of vehicles.

We measured traffic on the three lanes except the right-turn lane in the thermal images taken in August, one of which is shown in Figure 6(b). Figure 17 shows fluctuations of the numbers of vehicles classified in the three movement categories. We started measuring the numbers of vehicles when traffic lights were red. Thus, vehicle queues are composed in the whole measurement area, and almost all vehicles are stopped. After the traffic lights turn green, vehicles start and move, and the number of running vehicles increases. As shown in Figure 17, all vehicles maintain high speeds when the traffic lights are green. This shows that traffic flows are smooth. The number of fast moving vehicles decreases between the frames 91 and 101. If such decrease of traffic volume is occurred in an isolated traffic signal control, the traffic lights should be turned red.

This traffic monitoring can also detect traffic problems. If a vehicle stops suddenly on the outside lane when the traffic lights are green, the stopping vehicle is detected and an illegal parking can be estimated. If two or more vehicles stop suddenly when the traffic lights are green, the stopping vehicles are detected and a traffic accident can be estimated.

By monitoring the numbers of vehicles classified in the three movement categories, automatic traffic flow monitoring can be achieved. The traffic information is also useful to optimally control traffic signal lights.

## 6. Conclusions

First, we have explained briefly our previous method which detects vehicle positions and their movements by using thermal images obtained with an infrared thermal camera. Our experiments have shown that our previous method detects vehicles under three different environmental conditions, and the vehicle detection accuracy is 96.2%. Our previous method also detects vehicles robustly even under poor visibility conditions like snow and thick fog. Our previous method uses the windshield and its surroundings as the target of pattern recognition using the Viola-Jones detector. Some experiments in winter show that the vehicle detection accuracy decreases because the temperatures of many windshields approximate those of the exterior of the windshields.

Then, in this paper we have proposed a new vehicle detection method, and called it “our new method”. Our new method detects vehicles based on distinguishing the tires' thermal energy reflection area on the road surface from the other areas. We have done the experiments using three series of thermal images for which the vehicle detection accuracies of our previous method are low. Our new method detects 1,417 vehicles (92.8%) out of 1,527 vehicles, and the number of false detection is 52 in total. Therefore, by combining our two methods, high vehicle detection accuracies are maintained under various environmental conditions.

Finally, we have applied the traffic information obtained by our two methods to automatic traffic flow monitoring. An example of automatic traffic flow monitoring has been shown. By using the traffic information obtained by our two methods, we can achieve automatic traffic flow monitoring. We also expect to realize an optimal traffic signal control.

We have a plan to incorporate the following two algorithms into our new method in order to increase the current detection rate of 92.8% even further.

(1) To detect tires' thermal energy reflection area with higher accuracy, we use a detection line with a thickness of 2 pixels or more near a vehicle instead of the line with a thickness of 1 pixel. We will sensitively detect tires' thermal energy reflection area by increasing the thickness of the detection line.
(2) Each vehicle is tracked by effectively using spatio-temporal images as shown in Figure 15. By tracking each moving vehicle separately until it stops in a queue, and detecting the positions of occluded vehicles exactly in the queue, the detection rate for occluded vehicles will be increased.

As a future subject, we will investigate the work of our two methods under more severe condition such as ice slush and rain. In such a condition, we will confirm whether or not both temperatures of windshield and tires' thermal energy reflection area are as same as those of their surrounding areas. If such a situation occurs, and our two methods do not detect vehicles, we will improve the methods.
